# A systematic review protocol: social network analysis of tobacco use

**DOI:** 10.1186/2046-4053-3-85

**Published:** 2014-08-08

**Authors:** Raglan Maddox, Rachel Davey, Ray Lovett, Anke van der Sterren, Joan Corbett, Tom Cochrane

**Affiliations:** 1 Centre for Research and Action in Public Health, University of Canberra, University Drive, Canberra ACT 2606, Australia; 2 Australian Institute of Aboriginal and Torres Strait Islander Studies, Australian National University, GPO Box 553, Canberra ACT 2601, Australia; 3 Centre for Excellence in Indigenous Tobacco Control, University of Melbourne, 207 Bouverie Street, Melbourne VIC 3010, Australia; 4 Faculty of Health, University of Canberra, University Drive, Canberra ACT 2606, Australia

**Keywords:** Protocol, systematic review, tobacco use, smoking, social networks

## Abstract

**Background:**

Tobacco use is the single most preventable cause of death in the world. Evidence indicates that behaviours such as tobacco use can influence social networks, and that social network structures can influence behaviours. Social network analysis provides a set of analytic tools to undertake methodical analysis of social networks. We will undertake a systematic review to provide a comprehensive synthesis of the literature regarding social network analysis and tobacco use. The review will answer the following research questions: among participants who use tobacco, does social network structure/position influence tobacco use? Does tobacco use influence peer selection? Does peer selection influence tobacco use?

**Methods:**

We will follow the Preferred Reporting Items for Systemic Reviews and Meta-Analyses (PRISMA) guidelines and search the following databases for relevant articles: CINAHL (Cumulative Index to Nursing and Allied Health Literature); Informit Health Collection; PsycINFO; PubMed/MEDLINE; Scopus/Embase; Web of Science; and the Wiley Online Library. Keywords include tobacco; smoking; smokeless; cigarettes; cigar and ‘social network’ and reference lists of included articles will be hand searched. Studies will be included that provide descriptions of social network analysis of tobacco use.

Qualitative, quantitative and mixed method data that meets the inclusion criteria for the review, including methodological rigour, credibility and quality standards, will be synthesized using narrative synthesis. Results will be presented using outcome statistics that address each of the research questions.

**Discussion:**

This systematic review will provide a timely evidence base on the role of social network analysis of tobacco use, forming a basis for future research, policy and practice in this area. This systematic review will synthesise the evidence, supporting the hypothesis that social network structures can influence tobacco use. This will also include exploring the relationship between social network structure, social network position, peer selection, peer influence and tobacco use across all age groups, and across different demographics. The research will increase our understanding of social networks and their impact on tobacco use, informing policy and practice while highlighting gaps in the literature and areas for further research.

## Background

Tobacco use is a major public health concern due to significant associated health risks, such as cardiovascular disease, respiratory diseases and cancers [1-6]. As a result, tobacco use is the single most preventable cause of death in the world and is the most preventable cause of morbidity and mortality within Australia [7,8]. Tobacco use has spread globally throughout the developed and developing world [9]. It is well-documented that many cultural and socio-environmental factors influence tobacco use, with increased interest in the context of tobacco use within social networks [10-18]. Social network analysis provides a set of analytic tools to undertake methodical analysis of social networks; mapping, measuring and analysing relationships and exchange among interacting units, such as relationships between people, groups and organizations [19,20].

Evidence indicates that social network structures can influence behaviour and that behaviour can influence social networks, with normative and peer influences transmitted through network ties or relationships [11,21]. Peer associations can impact on behaviour, including smoking initiation and cessation [22-24]. In addition, tobacco use can assist to maintain and reinforce social relationships and kinship bonds [25-27]. Social network analysis is used in many disciplines to map, measure, characterise and investigate relationships and influences between people, groups, and organisations [18,28-31]. For example, economics, sociology, health and political science have all studied how real-life social networks can influence the spread of complex behaviour, such as tobacco and alcohol use, obesity, suicide prevention, organ donation registration and even political expression and voting behaviour [32-44]. A better understanding of these connections, relationships and influences through social networks analysis of tobacco use is required [11,18].

Undertaking the systematic review on social network analysis of tobacco use will improve our understanding of the interaction between social networks and smoking behaviour and attitudes across population groups. A systematic review by Seo and Huang [45] explored social network analysis in smoking behaviour, but only focused on adolescent cigarette smoking. This systematic review will build on the research by Seo and Huang [45], systematically consolidating and investigating social network analysis of tobacco use among all population groups. This review will contribute to the evidence base by highlighting and synthesising key learning, inconsistencies and any evidence-gaps that remain from research of social network analysis of tobacco use. This review could be used to further inform research, programmes and policies utilising social networks to address tobacco use.

This systematic review has not been registered with the International Prospective Register of Systematic Reviews (PROSPERO) as it does not meet the inclusion criterion. For example, PROSPERO requires a minimum of one outcome to be of direct patient or clinical relevance, which is outside the scope of this review.

### Research question/s

The systematic review will provide a comprehensive synthesis of the literature on social network analysis of tobacco use and summarise key findings and the nature of social network influences on tobacco use. The research questions include the following. 1) Does social network structure/position influence tobacco use? For example, are clique members, liaisons, and isolates more likely to use tobacco? 2) Does tobacco use influence peer selection? 3) Does peer selection influence tobacco use?

## Methods

This systematic review will follow the Preferred Reporting Items for Systemic Reviews and Meta-Analyses (PRISMA) guidelines [46].

### Criteria for considering studies

#### Study inclusion criteria

This review will include peer reviewed literature that is published in electronic databases. Studies must describe social network analysis, examining relationships between participants in regards to tobacco use [19,20].

##### Study design

Studies using quantitative, qualitative and mixed-methods approaches will be eligible for inclusion in order to obtain a comprehensive overview of the existing evidence base. This may include: case control; cohort; cross-sectional; experimental; and intervention designs with no restrictions. All relevant publications will be obtained in order to gain an overview of observational evidence and the influence of social structures on tobacco use.

##### Population

The sample must include tobacco users, but all genders, age groups and participants from any racial, ethnic, cultural or religious groups will be eligible for inclusion, regardless of location.

##### Intervention/exposure

Studies to be included must include a description of social network analysis of tobacco use, and may include observational data if the inclusion criterion is met. This will assist to provide an overview of existing evidence of the influence of social structures on tobacco use.

##### Outcomes

Studies will be included if they contain any outcomes related to tobacco use and social network structure or social network characteristics, such as social network positions. Based primarily on the need to address the research questions, we consider the main outcomes for the systematic review to be: tobacco use and social network position/s; peer selection in tobacco use; and peer influence in tobacco use.

### Study exclusion criteria

We will exclude any studies that are: not available in English; conference abstracts; books or grey literature. Furthermore, studies with inappropriate and/or insufficient quality will also be excluded from the analysis.

### Search strategy

In following the PRISMA guidelines [46] we will search the following databases for relevant articles: Cumulative Index to Nursing and Allied Health Literature (CINAHL); Informit Health Collection; PsycINFO; PubMed/MEDLINE; Scopus/Embase; Web of Science; and Wiley Online Library. Reference lists of included articles will also be hand-searched. The search will be undertaken by 31 May 2014 and include papers published between 1 January 2004 and 31 May 2014. Important keywords include: tobacco; smoking; smokeless; cigarettes; cigar and social network.

### Selection of studies

We will upload search results into EndNote and any duplicates will be removed. Prior to any screening, reviewers will undergo training to ensure a comprehensive understanding of the review question, the inclusion and exclusion criteria and a basic understanding of social network analysis of tobacco use. In the first round of screening, titles and abstracts will be screened for inclusion. Following preliminary screening, eligibility will be assessed through full-text screening. Eligibility for inclusion of papers will be assessed independently and in duplicate. At the title and abstract screening level, consensus must be reached with both reviewers in order to exclude an article; conflicts will be included. During full-text screening, disagreements will require resolution through consensus. If consensus cannot be achieved, a third reviewer will be called to make a decision. Quality monitoring of the screening process will be done by the first author (RM), who will randomly select 10% of the total articles for revision. Assistance from an independent reviewer will be used if necessary.

### Data extraction

A data extraction form will be developed and pilot-tested on a randomly selected subsection of studies. We will then amend the extraction form based on outcomes and feedback from the pilot testing phase. This will ensure a comprehensive data extraction process and optimise the usability of the extraction form. The data extraction form will ensure that the review extracts pertinent data to provide a comprehensive synthesis of the literature regarding social network analysis of tobacco use. The form will provide a mechanism to elicit data to describe key findings and the nature of social network influences on tobacco use. As per the PRISMA guidelines, data will be extracted from each study that meets the inclusion criteria, including: participants; interventions; comparisons; outcomes; study design (PICOS); social network analysis methodology, follow-up period; and funding source [45,46]. The extraction process will be completed independently. Quality monitoring of the extraction process will be done by the first author (RM), who will randomly select 10% of the included articles for revision. If there is a disagreement, this will be resolved through consensus. If a consensus cannot be reached, a third reviewer will make a decision.

If data are unclear, missing, or presented in a form that is unable to be reliably extracted, authors will be contacted to assist in the process. The corresponding author will be initially contacted by email, with the first author (if not the corresponding author) copied into all correspondence. If email addresses are not available, authors will be contacted by phone. Authors will be given seven days to respond to emails, after which they will be followed up with a phone call and an additional email. If no responses are received after an additional seven days, another phone call will be made to contact the author. Attempts to reach authors will occur for an additional seven days and if authors are unable to be contacted, the authors will be classified as uncontactable.

### Quality assessment and risk of bias

The quality of qualitative studies will be measured using the McMaster Quality Assessment Guidelines - Qualitative Form (Version 2.0) [47]. We will assess all studies for threats to internal and external validity, and develop an index of threats to validity.

### Analysis

Qualitative, quantitative and mixed-method data that meets the inclusion criteria for the review, including methodological rigour, credibility and quality standards as outlined, will be described and synthesized using narrative synthesis [48]. This approach is used to synthesise the evidence relevant to the research questions, summarising and explaining the findings of included studies. Results will be presented using a number of outcome statistics where possible to address each research question [48]. For example, in addressing the influence of social network structure/position on tobacco use, mean difference, relative risk, odds ratio, etcetera, could be used, if available, to identify differences in tobacco use among clique members, liaisons or isolates. This is expected to be similar in assessing if peer selection processes (nominating smokers within the social network) predict future tobacco use, or vice versa.

A standardised template for data extraction will be used by one reviewer, and will be checked by a second reviewer. Preliminary synthesis will develop an initial description of the included study results, incorporating outcome statistics against research questions where possible [48]. As patterns across study results emerge from the preliminary synthesis, reviewers will interrogate the data to identify and gain an understanding about any factors that may explain differences in direction and/or effect [48]. The narrative synthesis of evidence is expected to be reported in a table format, highlighting the key outcomes and addressing the research questions. In order to avoid potential biases, key points of difference between studies will be identified.

Meta-analysis and pooling of statistical results will not be undertaken in this instance.

## Discussion

A more detailed understanding of the influence of social networks and the importance of people’s social context in relation to tobacco use and the behavior-change process is required. We anticipate that the systematic review will synthesise evidence, including network characteristics, that social network structures can influence behaviour such as tobacco use. An expected strength of the review will be its ability to examine the relationship between social network structure, social network position and tobacco use across all age groups, and potentially different cultures and demographics. For example, do clique members, liaisons, and isolates influence tobacco use and does this vary by age or population group? The review will also examine peer selection and peer influence preceding tobacco use. The research will increase our understanding of social networks and the impact on tobacco use, informing policy and practice while highlighting gaps in the literature and areas for further research. This will assist researchers in exploring the influence of social networks on tobacco use and to examine if there is an association between social factors and being a smoker or a non-smoker.

Review findings will be disseminated in peer-reviewed publications and presentations, and made publicly available through appropriate mechanisms.

This protocol received input from the Australian Capital Territory (ACT) Aboriginal and Torres Strait Islander Tobacco Control Advisory Group.

### Limitations

This systematic review may not be generalizable across all population groups, such as minority groups and different age groups. In addition, the literature may not capture the holistic and dynamic nature of social networks, but their influence in relation to tobacco use, peer influence and peer selection at a point in time.

## Competing interests

The authors declare that they have no competing interests.

## Authors’ contributions

RM, conceived the protocol and drafted and finalised the manuscript. RD commented on the study protocol, revised the manuscript critically for important intellectual content and will be involved in the analysis and interpretation of data. RL has been involved in the preliminary discussion around the systematic review, contributing to the design of the review and will be involved in the analysis and interpretation of the systematic review data. RL was also involved in drafting the manuscript and revising it critically for important intellectual content. AVDS contributed in the design of the study and was involved in drafting the manuscript and revising it critically for important intellectual content. JC contributed to the design of the protocol, was involved in drafting the manuscript, and will be involved in the analysis and interpretation of the systematic review data. TC contributed to the study protocol, with particular input on analysis and interpretation of data. All authors read and approved the final manuscript.
